# Social Vulnerability Post-Incarceration: Analyzing Social Isolation and Loneliness Among Older Adults

**DOI:** 10.1093/geroni/igaf122.198

**Published:** 2025-12-31

**Authors:** Kenzie Latham-Mintus, Elizabeth Nelson, Melissa Garrido, Raya Kheirbek

**Affiliations:** Indiana University Indianapolis, Indianapolis, Indiana, United States; Indiana University Indianapolis, Indianapolis, Indiana, United States; Boston University School of Public Health, Boston, Massachusetts, United States; University of Maryland School of Medicine, Baltimore, Maryland, United States

## Abstract

**Background:**

This study examines how past incarceration affects social relationships in older adults.

**Objective:**

To explore the relationship between past incarceration and the quality and quantity of social relationships among older adults.

**Design, Setting, and Participants:**

Cross-sectional analysis of 13,023 Americans aged 50 and older from the Health and Retirement Study (HRS). Participants self-reported their history of incarceration.

**Main Outcomes and Measures:**

Multivariable logistic regression determined the odds of lacking close ties, low social integration, and high loneliness, while linear regression evaluated the relationship between incarceration history and the number of close ties, controlling for demographic factors.

**Results:**

Among 13,768 respondents, 992 (7.2%) reported a history of incarceration. They were more likely to be unmarried or unpartnered (aOR: 1.34, 95% CI: 1.11-1.61), rate spousal closeness lower (aOR: 0.59, 95% CI: 0.50-0.70), and have no close relationships with children (aOR: 1.42, 95% CI: 1.19-1.69), family (aOR: 1.28, 95% CI: 1.06-1.54), or friends (aOR: 1.21, 95% CI: 1.01-1.44). They also had higher odds of low participation (aOR: 1.42, 95% CI 1:18, 1.71), low cohesion (aOR: 1.35, 95% CI 1.13, 1.60), and high loneliness (aOR: 1.67, 95% 1.43, 1.94). Linear regression revealed a similar pattern of social vulnerability across all social measures.

**Conclusions:**

Older adults with a history of incarceration are at increased risk of social isolation and loneliness. Targeted interventions could improve their social integration and well-being.

